# Risk factors for vertebral fractures in postmenopausal women: a meta-analysis

**DOI:** 10.3389/fmed.2026.1888923

**Published:** 2026-08-03

**Authors:** Yi He, Jing Zhang

**Affiliations:** Nanbu County People's Hospital, Nanchong, China

**Keywords:** meta-analysis, postmenopausal, risk factors, systematic review, vertebral fractures

## Abstract

**Background:**

Vertebral fractures are the most common osteoporotic fractures in postmenopausal women and are strongly associated with higher morbidity, lower quality of life, and higher risk of death. Identifying risk factors for vertebral fractures in postmenopausal women is essential for early prevention and intervention. The aim of this study was to systematically assess and quantify the risk factors for vertebral fractures in postmenopausal women by meta-analysis.

**Methods:**

We conducted a comprehensive search in PubMed, Embase, Web of Science, and Cochrane Library databases to include all observational studies reporting risk factors for vertebral fractures in postmenopausal women. The search was conducted from the date of database creation to April 20, 2025, Study screening, data extraction, and quality assessment were performed by two independent reviewers, and study quality was assessed using the Newcastle-Ottawa scale. Data were analyzed using Stata15 software.

**Results:**

A total of 11 studies involving 7,605 participants were included. The meta-analysis showed that advanced age [odds ratio (OR) = 1.07, 95% confidence interval (CI): 1.03–1.12], higher body mass index (BMI; OR = 1.07, 95% CI: 1.01–1.14), previous fracture (OR = 1.78, 95% CI: 1.29–2.45), low lumbar-spine bone mineral density (BMD; OR = 1.55, 95% CI: 1.27–1.89), glucocorticoid use (OR = 2.34, 95% CI: 1.34–4.09), and rheumatoid arthritis (OR = 2.99, 95% CI: 1.38–6.45) were associated with a higher likelihood of vertebral fracture in postmenopausal women.

**Conclusion:**

This meta-analysis showed that risk factors for vertebral fracture in postmenopausal women include age over 60 years, higher body mass index, history of previous fracture, low lumbar bone density, glucocorticoid use, and rheumatoid arthritis. These factors significantly increase the risk of vertebral fractures. Therefore, early screening and intervention in these high-risk groups is warranted to reduce the incidence of vertebral fractures and to provide more individualized prevention and treatment strategies.

## Background

In modern societies, aging is gradually becoming an important public health challenge worldwide as people live longer (1). Osteoporosis is one of the most common metabolic bone diseases in the elderly population and is especially prominent in postmenopausal women (2). The main manifestations of osteoporosis are reduced bone density, bone fragility, and susceptibility to fracture (3). According to the World Health Organization (WHO) definition, osteoporosis is defined as a significant increase in the risk of fracture when bone density is lower than normal (4). Among them, vertebral fracture, as one of the typical manifestations of osteoporosis, not only brings severe physical pain to patients, but also may lead to dysfunction, disability, or even death, which seriously affects patients' quality of life and socioeconomic burden (5). Vertebral fractures are particularly common in postmenopausal women, especially in older women, and spinal compression fractures due to osteoporosis are frequent. These fractures not only increase the risk of the patient's disease, but also aggravate the complications associated with osteoporosis (spinal deformity, bone pain) (6).

Menopause is a physiologic process experienced by women that marks the decline of ovarian function and a significant decrease in estrogen levels. The decrease in estrogen has a particularly significant effect on bone, as estrogen plays an important role in bone metabolism, inhibiting bone resorption and promoting bone formation (7, 8). As a result, postmenopausal women are susceptible to decreased bone density and increased bone fragility, which can lead to a significant increase in fracture risk (9).

Previous studies (10–12) have identified advanced age, low bone mineral density, previous fracture, glucocorticoid exposure, rheumatoid arthritis, and several anthropometric and lifestyle characteristics as potential risk factors for vertebral fracture in postmenopausal women. However, the reported associations have not been consistent across populations and study designs. Differences in participant characteristics, definitions and assessment methods for vertebral fracture, follow-up duration, exposure thresholds, and adjustment for confounding factors have resulted in uncertainty regarding the magnitude of these associations. Furthermore, the available evidence has not been comprehensively quantified in a systematic review focusing specifically on postmenopausal women. Therefore, this systematic review and meta-analysis aimed to identify and quantify factors associated with vertebral fracture in postmenopausal women and to evaluate the heterogeneity and robustness of the available evidence.

## Methods

This systematic evaluation and meta-analysis will strictly follow the PRISMA (Preferred Reporting Items for Systematic Reviews and Meta-Analyses) guidelines (13). The systemic review was supported by the online PROSPERO international prospective register of systemic reviews of the National Institute for Health Research, registration number: (CRD420251013838).

## Literature search

We conducted a comprehensive search of all observational studies reporting risk factors for vertebral fractures in postmenopausal women in PubMed, Embase, Web of Science, and Cochrane Library databases. The databases were searched from their inception to April 20, 2025, with no lower publication-date restriction. The search terms were vertebral fractures; postmenopausal; risk factors, and the specific search strategy is described in Supplementary Table S1.

## Inclusion and exclusion criteria

According to the PICO framework, the inclusion criteria for this study were: (1) Population: the study population should be postmenopausal women, women aged 50 years and older. All subjects should be naturally menopausal or surgically menopausal women and should not have serious comorbidities or diseases affecting bone metabolism (cancer, heart disease). (2) Exposure: occurrence vertebral fractures. (3) Outcome: the primary outcome was a risk factor for vertebral fracture (the outcome was the presence or occurrence of vertebral fracture). For cross-sectional and case-control studies, the outcome was defined as prevalent vertebral fracture assessed at enrollment or during the retrospective assessment period specified by the original study. For cohort studies, the outcome was incident vertebral fracture occurring during the reported follow-up period. We extracted the definition, assessment method, and assessment or follow-up time frame for vertebral fracture from each study. (4) Study design: the study mainly included case-control, cohort study, and cross-sectional study.

## Exclusion criteria

Population: studies involving premenopausal women, men, or mixed populations from which data for postmenopausal women could not be extracted separately; studies involving women with serious comorbidities, such as cancer, diabetes mellitus, or heart disease, or conditions substantially affecting bone metabolism.

Exposure and outcome: studies that did not evaluate potential risk factors for vertebral fracture or did not report vertebral fracture outcomes.

Study design: case reports, case series, reviews, editorials, letters without original data, conference abstracts with insufficient information, and or animal studies (case reports and case series were excluded because they lacked an appropriate comparison group and could not provide reliable comparative effect estimates. Reviews, editorials, letters without original data, laboratory or animal studies, and conference abstracts with insufficient information were excluded because they did not provide original epidemiological data suitable for quantitative synthesis).

Data availability: studies that did not report an effect estimate or sufficient data from which an effect estimate, and its 95% confidence interval could be calculated.

## Study selection

During the literature screening process, two researchers independently used EndNote 21 software to initially screen the literature obtained from the search, first through the titles and abstracts, and then to exclude literature that clearly did not meet the inclusion criteria. Subsequently, the remaining literature was reviewed by reading the full text in its entirety to further determine whether it met the inclusion and exclusion criteria. In case of disagreement between the two researchers during the screening process, it would be resolved through discussion and negotiation; if the negotiation still failed to reach a consensus, a third researcher would be invited to adjudicate to ensure the objectivity and consistency of the screening process.

## Data extractions

This study was conducted by two researchers who independently extracted relevant data from the eligible literature using an Excel sheet based on the inclusion criteria. The extraction included the basic information of the study (first author, year of publication, country and type of study), the basic characteristics of the study population (sample size, gender, and mean age), the statistical model used in the regression analysis (multifactorial regression, univariate analysis), and the effect sizes of the exposure factors and the outcome indexes [odds ratio (OR) and the corresponding 95% confidence interval (CI)]. In the process of data extraction, if two investigators disagreed on the data, it would be resolved through negotiation, and if no agreement could be reached, a third investigator would adjudicate to ensure the accuracy and consistency of data extraction.

## Quality evaluation

The methodological quality of the included studies was assessed using tools appropriate to each study design. Case-control and cohort studies were evaluated using the Newcastle–Ottawa Scale (NOS) (14), which assessed study quality across three domains: selection of participants, comparability of study groups, and ascertainment of exposure or outcome. Cross-sectional studies were evaluated using the Agency for Healthcare Research and Quality (AHRQ) checklist (15), which assessed the study design, representativeness of the sample, data-collection procedures, and measurement of exposures and outcomes. Two reviewers independently assessed the methodological quality of each study. Any disagreement was resolved through discussion, and a third reviewer was consulted when consensus could not be reached.

## Statistical analysis

In this study, the OR and the corresponding 95% CI of each included study were combined using Stata version 15.0 (StataCorp LLC, College Station, TX, United States), for dichotomous factors, the OR compared participants with the factor of interest with the relevant reference group—for example, previous fracture vs. no previous fracture, glucocorticoid use vs. no use, and rheumatoid arthritis vs. no rheumatoid arthritis. For continuous factors, the unit represented by the OR, such as per 1-year increase in age, per 1-kg/m^2^ increase in BMI, or per 1-standard-deviation decrease in lumbar-spine BMD, was extracted and reported. First, for each study, we extracted the corresponding effect size OR and its 95% confidence interval. To combine these ORs, we pooled them using a random effects model, which can account for heterogeneity between studies, that is, variability in effect sizes across studies. ORs and 95% CIs were calculated for each study and combined into an overall effect size. Heterogeneity of the model was assessed by the *I*^2^ statistic; if the *I*^2^ was greater than 50%, it was considered that there was a high degree of heterogeneity and that the sources of heterogeneity needed to be further explored. For each factor reported by at least three studies, robustness was assessed using a leave-one-out sensitivity analysis, in which the meta-analysis was repeated after sequential exclusion of each study. In addition, funnel plots and Egger's test were used to assess the likelihood of publication bias. If bias exists, it may have an impact on the interpretation of the results. The combined effect sizes will be reported as ORs and their 95% CIs to allow for interpretation of results and statistical inference.

## Results

### Literature screening results

**Figure 1:**
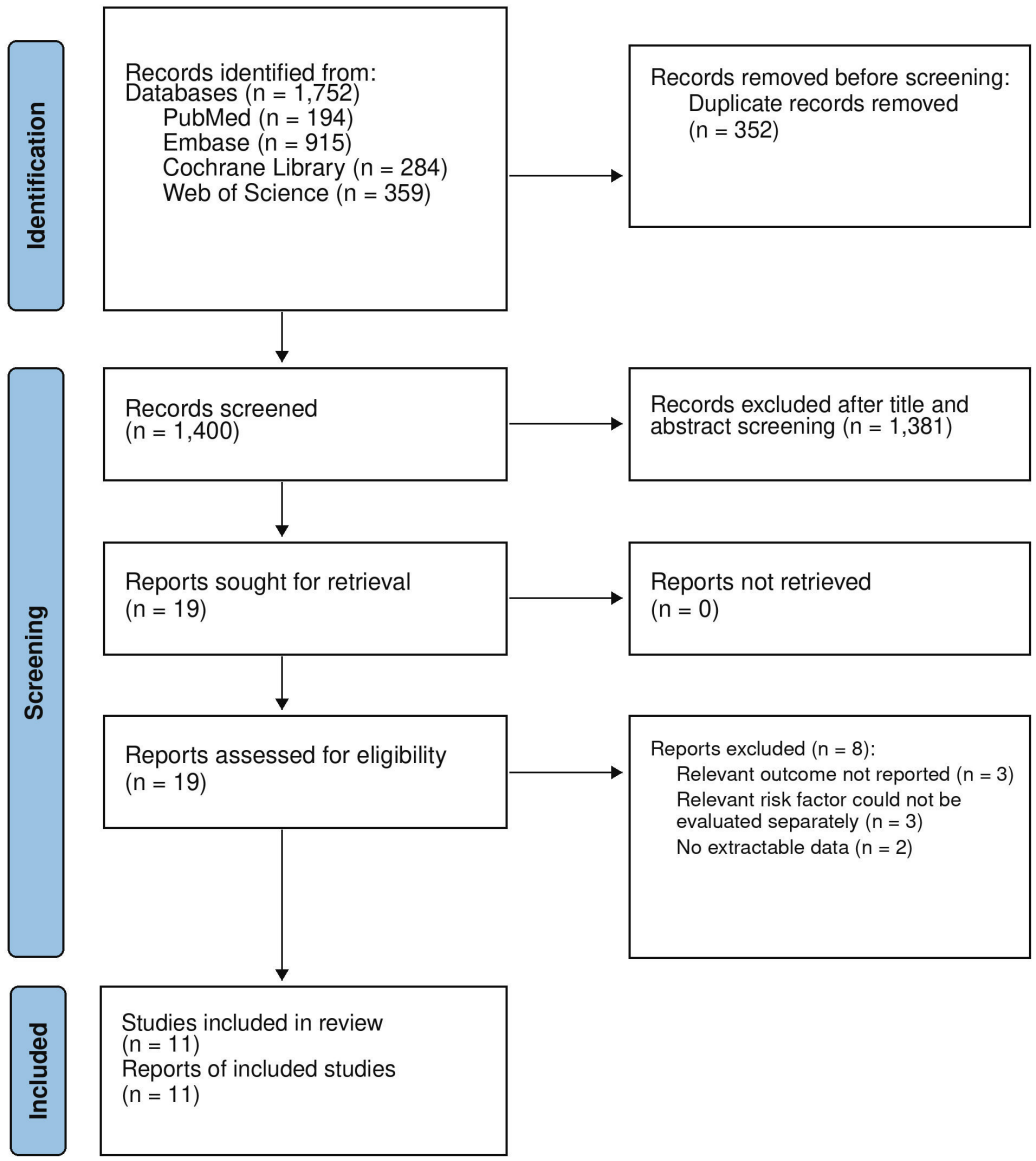
Literature search flow chart.

The study identification and selection process is presented in the PRISMA 2020 flow diagram (Figure 1). A total of 1,752 records were identified from PubMed (*n* = 194), Embase (*n* = 915), the Cochrane Library (*n* = 284), and Web of Science (*n* = 359). After 352 duplicate records were removed, 1,400 records underwent title and abstract screening, of which 1,381 were excluded. The full texts of the remaining 19 reports were retrieved and assessed for eligibility. Eight reports were excluded because they did not report a relevant outcome (*n* = 3), involved combined interventions or did not allow the relevant factor to be evaluated separately (*n* = 3), or lacked extractable data (*n* = 2). Finally, and 11 studies (16–26) were included in the final meta-analysis.

### Basic characteristics of literature

There was a total of 11 studies (*N* = 7,605), of which 6 studies (16, 17, 23–26) were cross-sectional, 4 studies (18–21) were cohort studies and 1 study (22) was a case-control study. The prevalence of vertebral fractures was 21.3% (1,617/7,605) with an age distribution of 59.9–70.2 years, most of the studies used multivariate logistic regression. The basic characteristics of the specific included studies are tabulated in Table 1.

**Table 1:** Basic characteristic table.

Study	Year	Country	Study design	Sample size	Number of vertebral fractures	Mean age (years)	Regression model
Arboleya	2010	Asturias	Cross-section	287	145	64.4	Not reported
Cano	2016	Spain	Cross-sectional	728	284	62.1	Multivariate logistic regression
Cheng	2024	China	Cohort study	682	100	70.18	Multivariate logistic regression
Ho-Pham	2009	Vietnam	Cross-sectional	209	48	62	Multivariate logistic regression
Jiajue	2019	China	Cohort study	982	43	62.1	Multivariate logistic regression
Kuroda	2024	Japan	Cohort study	1480	473	65.5	Multivariate logistic regression
Roux	2009	France	Case-control	1211	49	65.8	Multivariate logistic regression
Samakkarnthai	2024	Thailand	Cross-sectional	435	140	64.2	Multivariate logistic regression
Sanfelix	2013	Spain	Cross-sectional	804	126	64	Multivariate logistic regression
Wattanachanya	2021	Thailand	Cross-sectional	490	142	59.9	Multivariate logistic regression
Wu	2025	China	Cohort study	297	67	65.4	Multivariate logistic regression

### Quality assessment results

Table 2 included case-control studies, cohort studies and cross-sectional studies were assessed in this study by using different quality assessment tools. For case-control studies (22), most of the study designs, selection of case and control groups, and identification of exposure factors were well controlled, however, some of the studies had nonresponse issues. Cohort studies (18, 20) generally received high ratings and showed high quality especially in the selection of exposed and unexposed groups, and assessment of outcomes, but some of the studies had room for further optimization in design and statistical analysis. Cross-sectional studies (16) performed well on most of the indicators, especially in terms of data sources and inclusion criteria, but there were still some unclear parts, such as control of assessors' subjective factors and specification of reasons for patient exclusion.

Table 2Quality assessment results.

**Table:** 

References	Is the case definition adequate?	Representativeness of the cases	Determination of control group	Definition of Controls	Comparability of cases and controls based on the design or analysis	Ascertainment of exposure	Same method of ascertainment for cases and controls	Non response	Total scores
Case control									
Roux et al. (22 )	*	*	*	*	*	*	*	*	8
**References**	**Representativeness of the exposed group**	**Selection of non-exposed groups**	**Determination of exposure factors**	**Identification of outcome indicators not yet to be observed at study entry**	**Comparability of exposed and unexposed groups considered in design and statistical analysis**	**Design and statistical analysis**	**Adequacy of the study's evaluation of the outcome**	**Adequacy of follow-up in exposed and unexposed groups**	**Total scores**
Cohort study									
Cheng et al. (18)	*	*	*	*	^**^	*	*	*	9
Ho-Pham et al. (19)	*	*	*	*	*	*	*	*	8
Jiajue et al. (20)	*	*	*	*	^**^	*	*	*	9
Kuroda et al. (21)	*	*	*	*	^**^	*	*	*	9

**Table:** 

Study	#1	#2	#3	#4	#5	#6	#7	#8	#9	#10	#11
Cross-section											
Arboleya et al. (16)	Yes	Yes	Yes	Yes	Yes	Unclear	Yes	Yes	Yes	Unclear	Unclear
Cano et al. (17)	Yes	Yes	Yes	Yes	Yes	Unclear	Yes	Yes	Yes	Unclear	Unclear
Cheng et al. (18)	Yes	Yes	Yes	Yes	Yes	Unclear	Yes	Yes	Yes	Unclear	Unclear
Ho-Pham et al. (19)	Yes	Yes	Yes	Yes	Unclear	Yes	Yes	Yes	Yes	Yes	Yes
Jiajue et al. (20)	Yes	Yes	Yes	Yes	Unclear	Yes	Yes	Yes	Yes	Yes	Yes
Kuroda et al. (21)	Yes	Yes	Yes	Yes	Unclear	Yes	Yes	Yes	Yes	Yes	Yes

#1 Is the source of the data identified (survey, literature review)?

#2 Are the inclusion and exclusion criteria for exposed and unexposed groups (cases and controls) listed or referred to previous publications?

#3 Is the time for identifying patients given?

#4 If not population origin, were the subjects continuous?

#5 Does the subjective factor of the evaluator cover up other aspects of the research object?

#6 describes any quality assurance assessments performed (e.g., testing/retesting of the primary outcome).

#7 reasons for excluding any patients from the analysis are explained;

#8 Measures to evaluate and/or control confounding factors;

#9 if possible, explain how missing data were handled in the analysis;

#10 summarizing the patient response rate and completeness of data collection;

#11 If follow-up is available, identify the percentage of patients with expected incomplete data or follow-up results.

^*^one score, ^**^two scores.

## Meta analysis results

### Age>60 years

**Figure 2:**
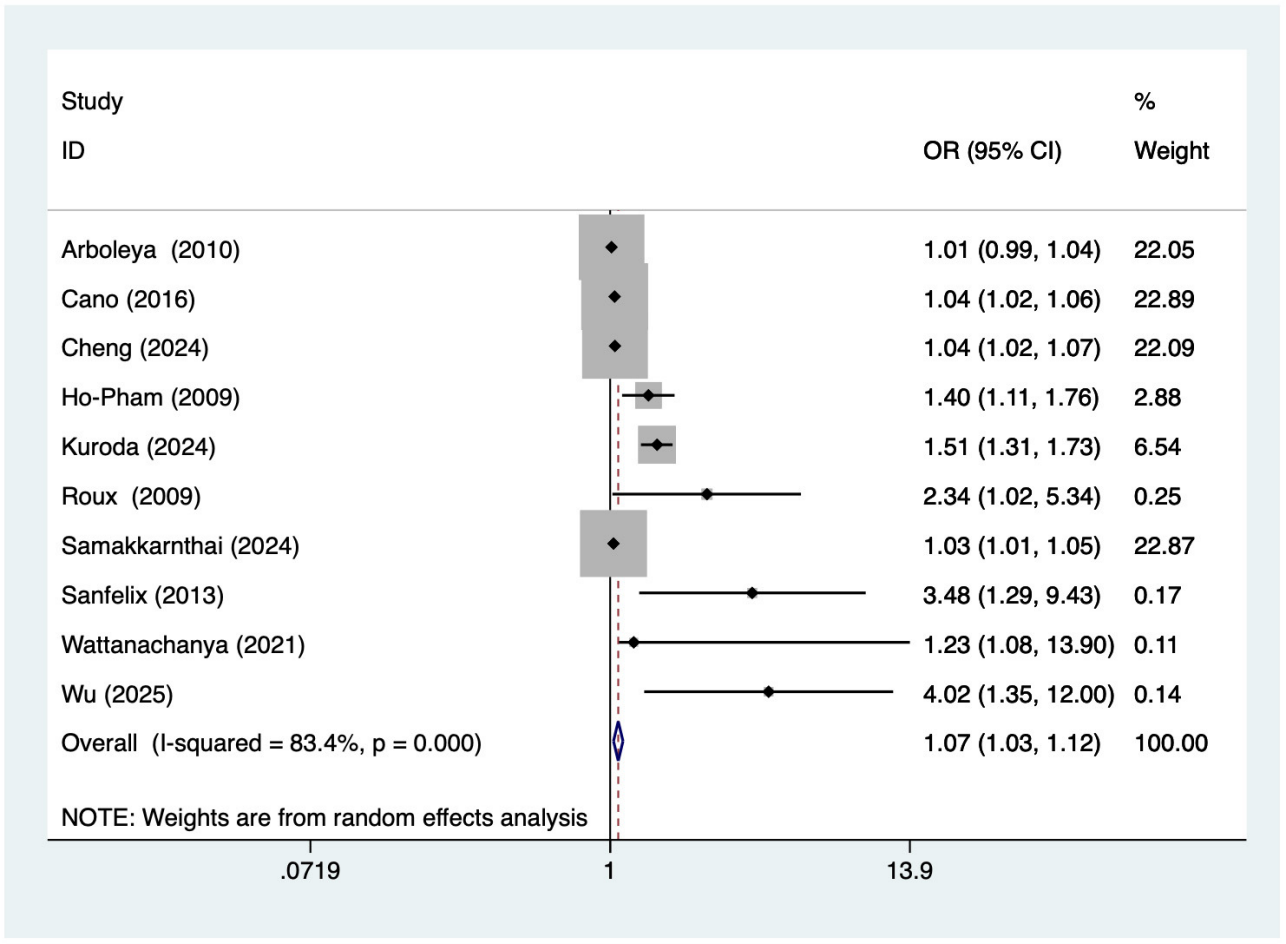
Forest plot of meta-analysis for Age>60 years.

Ten articles mentioned age >60 years, heterogeneity test (*I*^2^ = 83.4%, *P* = 0.001), and random effects model was used for the analysis, and the results of the analysis (Figure 2) suggested that age >60 years was a risk factor for vertebral fractures in women with late menstruation [OR = 1.07, 95%CI (1.03, 1.12)], and due to the high degree of heterogeneity, literature-by-literature exclusion was used to conduct the sensitivity analysis, and the results of the analysis (Supplementary Figure S1) suggested that the sensitivity was small and the results of the analysis were relatively stable.

### Body mass index (BMI) ≥25 kg/m^2^

**Figure 3:**
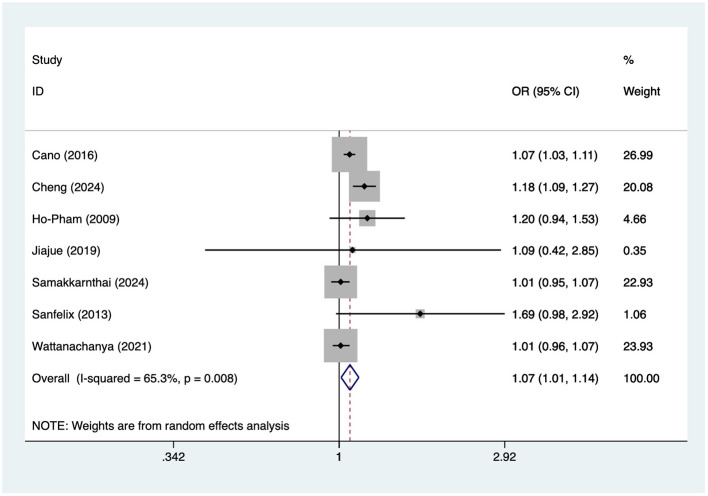
Forest plot of meta-analysis for BMI≥25 kg/m^2^.

Seven articles mentioned BMI≥25 kg/m^2^, heterogeneity test (*I*^2^ = 65.3%, *P* = 0.008), and random effects model was used for the analysis, and the results of the analysis (Figure 3) suggested that BMI≥25 kg/m^2^ was a risk factor for vertebral fractures in women with late menstruation [OR = 1.07, 95%CI (1.01, 1.14)], and due to the high degree of heterogeneity, literature-by-literature exclusion was used to conduct the sensitivity analysis, and the results of the analysis (Supplementary Figure S2) suggested that the sensitivity was small and the results of the analysis were relatively stable.

### Previous fractures

**Figure 4:**
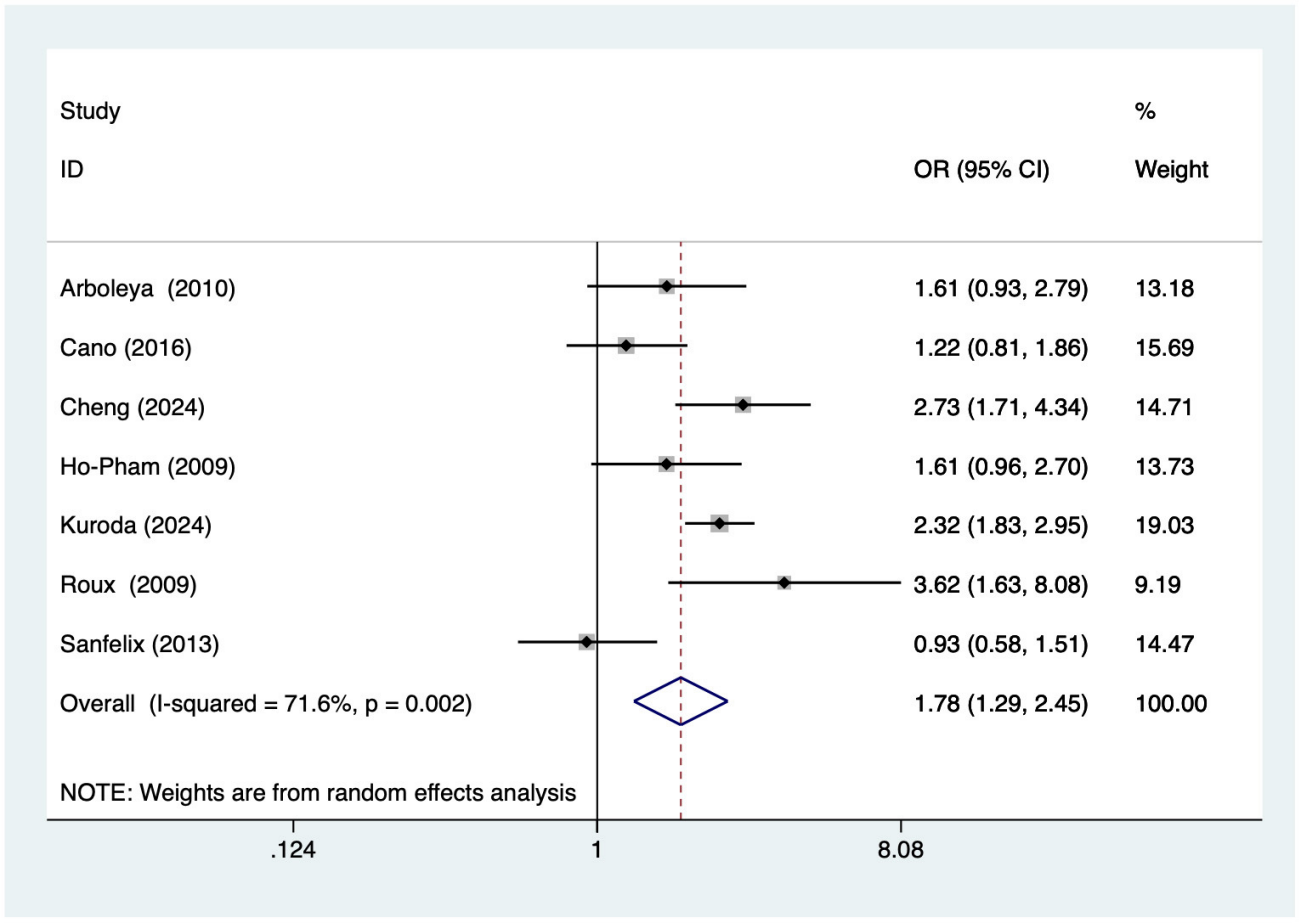
Forest plot of meta-analysis for previous fractures.

Seven articles mentioned previous fractures, heterogeneity test (*I*^2^ = 71.6%, *P* = 0.002), and random effects model was used for the analysis, and the results of the analysis (Figure 4) suggested that previous fractures was a risk factor for vertebral fractures in women with late menstruation [OR = 1.78, 95%CI (1.29, 2.45)], and due to the high degree of heterogeneity, literature-by-literature exclusion was used to conduct the sensitivity analysis, and the results of the analysis (Supplementary Figure S3) suggested that the sensitivity was small and the results of the analysis were relatively stable.

### Low lumbar BMD

**Figure 5:**
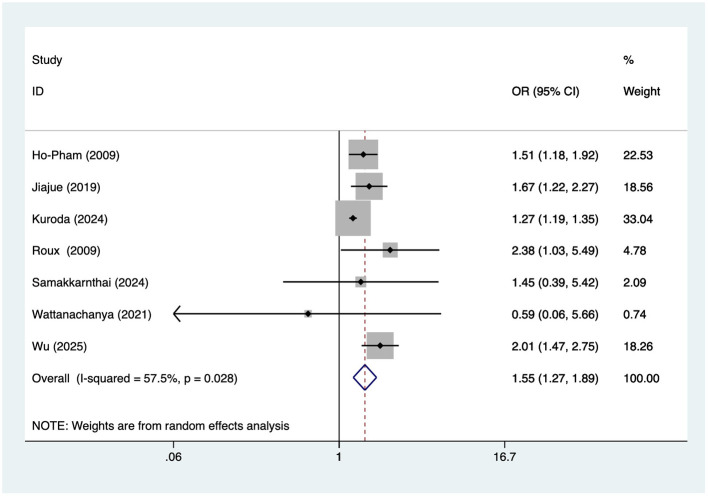
Forest plot of meta-analysis for low lumbar BMD.

Seven articles mentioned low lumbar BMD, heterogeneity test (*I*^2^ = 57.5%, *P* = 0.028), and random effects model was used for the analysis, and the results of the analysis (Figure 5) suggested that low lumbar BMD was a risk factor for vertebral fractures in women with late menstruation [OR = 1.55, 95%CI (1.27, 1.89)], and due to the high degree of heterogeneity, literature-by-literature exclusion was used to conduct the sensitivity analysis, and the results of the analysis (Supplementary Figure S4) suggested that the sensitivity was small and the results of the analysis were relatively stable.

### Glucocorticoid used

**Figure 6:**
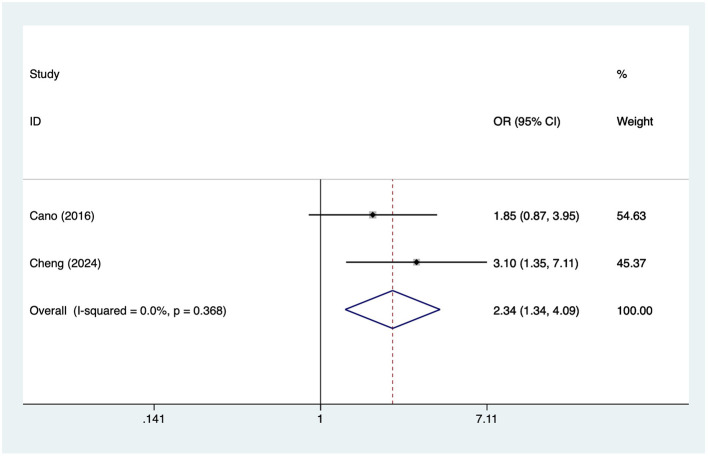
Forest plot of meta-analysis for glucocorticoid.

Two articles mentioned glucocorticoid used, heterogeneity test (*I*^2^ = 0%, *P* = 0.368), and fixed effects model was used for the analysis, and the results of the analysis (Figure 6) suggested that glucocorticoid used was a risk factor for vertebral fractures in women with late menstruation [OR = 2.34, 95%CI (1.34, 4.09)].

### Rheumatoid arthritis

**Figure 7:**
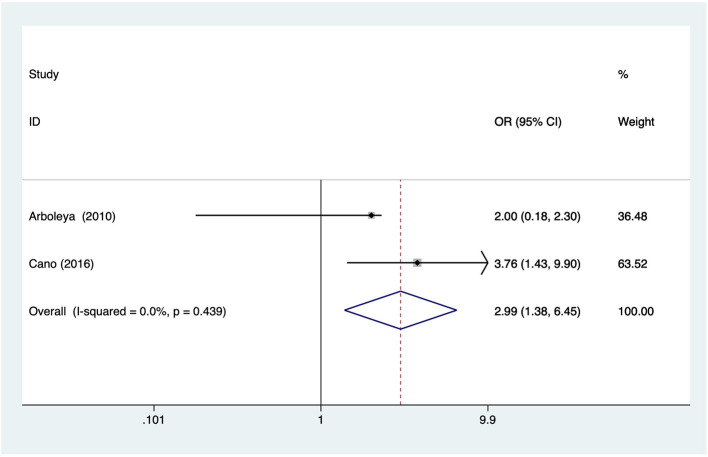
Forest plot of meta-analysis for rheumatoid arthritis.

Two articles mentioned rheumatoid arthritis, heterogeneity test (*I*^2^ = 0%, *P* = 0.368), and fixed effects model was used for the analysis, and the results of the analysis (Figure 7) suggested that rheumatoid arthritis was a risk factor for vertebral fractures in women with late menstruation [OR = 2.99, 95%CI (1.38, 6.45)].

### Publication bias

In the current study, funnel plots (Supplementary Figures S5-S8) and Egger's test were used to detect publication bias for outcomes with >2 included studies, and the results suggested that age >60 years (*P* = 0.001), both sides of the funnel plots were asymmetric, and the likelihood of publication bias was higher. BMI ≥25 kg/m^2^ (*P* = 0.346), Previous fractures (*P* = 0.623), Low Lumbar BMD (*P* = 0.133) were less likely to have publication bias.

## Discussion

In this study, we investigated the effects of age, BMI, previous fracture, low lumbar spine bone density, glucocorticoid use and rheumatoid arthritis on spinal fractures in late menopausal women through a meta-analysis of 11 studies. The results of the analysis showed that age >60 years, BMI ≥25 kg/m^2^, previous fracture, low lumbar spine bone density, glucocorticoid use, and rheumatoid arthritis were all important risk factors for spinal fractures. These factors provide a valid basis for clinical risk assessment and help to identify high-risk groups and take intervention measures.

Many studies have demonstrated that age greater than 60 years is an independent risk factor for spinal fractures. For example, Bolland et al. (27) found through a prospective cohort study that the incidence of fracture increased significantly with age, especially among postmenopausal women. Similarly, Kozaki et al. (28) reported a significant association between age and fracture risk. With increasing age, there is a gradual decrease in bone mineral density, deterioration of bone microarchitecture, and increased fracture risk. Especially in women after menopause, estrogen levels decline, bone resorption increases, and bone formation decreases, thus accelerating the process of osteoporosis. In addition, as we age, our muscle strength weakens, leading to an increased risk of falling, which in turn increases the likelihood of fracture (29). Therefore, for the elderly, regular bone density testing, calcium and vitamin D supplementation, as well as the use of bone-protecting medications (bisphosphonates) under the direction of a physician are important tools to reduce the incidence of fractures. Obesity may increase the risk of fractures in several ways. On the one hand, heavier body weight increases loads on areas such as the spine, hips, and knees, leading to excessive stress on the bones and thus increasing the risk of fracture (30). On the other hand, the chronic low-grade inflammatory response (increased C-reactive protein levels) that often accompanies obesity affects bone metabolism, decreasing bone quality and increasing bone resorption (31). In addition, obesity may affect the microarchitecture of the skeleton, leading to osteoporosis, and Kupisz et al. (32) noted that obese women have a higher risk of fracture than normal-weight women, especially among postmenopausal women. Weight control is the key to preventing obesity-related fractures. A healthy diet, avoidance of high-calorie foods, and regular exercise are crucial for maintaining a healthy body weight. In addition, obese patients should be concerned about bone density testing and use bone-protective medications to reduce fracture risk if necessary. The occurrence of a previous fracture may lead to changes in the bone structure, such as a further decrease in bone density or destruction of the bone microstructure, making the bone more susceptible to new fractures (33). In addition, previous fractures can lead to reduced activity and muscle strength, increasing the risk of falls, which is also an important factor in the occurrence of secondary fractures. This is also as found in a study by Kanis et al. (34), who found that individuals with a previous fracture had more than twice the risk of a subsequent fracture compared with those without a previous fracture. For patients with a history of fracture, regular bone density examinations should be performed to assess the risk of fracture, timely bone-protecting treatments should be administered, and post-fracture rehabilitation should be strengthened to promote bone healing, enhance muscle strength, and reduce the likelihood of re-fracture.

Low lumbar BMD indicates a decrease in bone quality, and decreased bone density directly affects bone strength and increases the risk of fracture. Additionally, the lumbar spine is one of the common sites of osteoporosis-induced fractures, and low lumbar BMD may reflect gaps in overall bone health (35). Garcia et al. (36) found through a multicenter study that low lumbar BMD was an independent risk factor for spinal fractures. For these patients, adequate calcium and vitamin D supplementation should be provided to prevent further bone loss. Several studies have shown that long-term use of glucocorticoids is strongly associated with the development of osteoporosis and fractures. (40) noted that glucocorticoids are a known causative factor for osteoporosis, and that patients on long-term glucocorticoid use are at a significantly increased risk of fracture. Glucocorticoids disrupt bone metabolism by inhibiting bone formation and increasing bone resorption, leading to decreased bone density. In addition, glucocorticoids affect calcium absorption and bone mineralization, which in turn makes bones weaker. Long-term glucocorticoid use may also affect muscle health and increase the risk of falls (37). For patients using glucocorticoids for a long period of time, bone density should be checked regularly, and if necessary, bone density tests and bone-protecting medications should be administered. Patients with rheumatoid arthritis often have chronic inflammation, and inflammatory mediators such as tumor necrosis factor and interleukin-6 affect bone metabolism and promote bone resorption. In addition, patients with rheumatoid arthritis may be on glucocorticoids or other immunosuppressive agents for long periods of time, which further increases the risk of fracture (38). Kanis et al. (39) found that patients with rheumatoid arthritis are more prone to osteoporosis and fractures than the general population. Patients with rheumatoid arthritis should focus on osteoporosis prevention and treatment along with treatment of the underlying disease, as well as moderate exercise to reduce fracture risk.

## Clinical significance

The results of this study have important implications for clinical practice. Firstly, it helps clinicians to identify high-risk patient groups, especially the group of late menopausal women, who have a significantly higher incidence of fractures. Early identification of these risk factors and appropriate interventions (bone density testing, lifestyle improvement, and pharmacological treatment) can significantly reduce the incidence of spinal fractures. In addition, this study provides an important basis for the development of individualized fracture prevention strategies, especially for women who are older, heavier, have a history of previous fracture or use glucocorticoids.

## Strengths and limitations

The strength of this study lies in the use of meta-analysis, which integrates the results of several studies and can provide more reliable statistical conclusions. Different types of studies (cross-sectional, cohort, and case-control studies) were included in the study and sensitivity analyses were conducted to enhance the stability and general applicability of the findings.

However, there are some limitations of this study. Firstly, despite the large number of included studies, there was a high degree of heterogeneity, which may have resulted in inconsistent results due to different regions, study designs and sample characteristics. Second, some of the included studies suffered from publication bias, which may have affected the objectivity of the results. In addition, this study relied mainly on observational studies, which could not completely exclude the influence of confounding factors. Therefore, higher-quality prospective studies are needed in the future to further validate the relationship between these risk factors and spinal fractures and to explore more individualized intervention strategies.

## Conclusion

In this study, a systematic evaluation and meta-analysis of 11 studies identified several significant risk factors associated with spinal fractures in postmenopausal women, including age >60 years, BMI ≥25 kg/m^2^, history of previous fracture, low lumbar spine bone mineral density, glucocorticoid use, and rheumatoid arthritis. However, due to the limitations of the study, further prospective studies are still needed to verify the generalizability and mechanism of action of these risk factors in different populations. Future studies should focus on how to optimize the existing fracture prediction models and further explore more potential risk factors and their mechanisms, with the aim of providing a more comprehensive and precise guidance for fracture prevention.

## Data Availability

The original contributions presented in the study are included in the article/Supplementary material, further inquiries can be directed to the corresponding author.
