# Supplementary material for: Risk factors for vertebral fractures in postmenopausal women: a meta-analysis
Source: Front Med (Lausanne). 2026 Aug 3;13:1888923. doi: 10.3389/fmed.2026.1888923 (PMC13477985; doi:10.3389/fmed.2026.1888923)
Supplement: Supplementary file 1 [file Data_Sheet_1.docx]

Table S1 search strategy

((("Postmenopause"[Mesh]) OR (((((((((Postmenopause[Title/Abstract]) OR (Post-menopausal Period[Title/Abstract])) OR (Period, Post-menopausal[Title/Abstract])) OR (Post menopausal Period[Title/Abstract])) OR (Post-Menopause[Title/Abstract])) OR (Post Menopause[Title/Abstract])) OR (Post-Menopauses[Title/Abstract])) OR (Postmenopausal Period[Title/Abstract])) OR (Period, Postmenopausal[Title/Abstract]))) AND (("Spinal Fractures"[Mesh]) OR ((((((((((((Spinal Fractures[Title/Abstract]) OR (Fracture, Spinal[Title/Abstract]))) OR )) OR (Fractures, Spinal[Title/Abstract])) OR (Spinal Fracture[Title/Abstract])) OR (Hangman Fracture[Title/Abstract])) OR (Fracture, Hangman[Title/Abstract])) OR (Hangman's Fracture[Title/Abstract])) OR (Fracture, Hangman's[Title/Abstract])) OR (Hangmans Fracture[Title/Abstract])) OR (Vertebral fractures[Title/Abstract])))) AND (("Risk Factors"[Mesh]) OR (((((((((((((((((((Risk Factors[Title/Abstract]) OR (Factor, Risk[Title/Abstract])) OR (Risk Factor[Title/Abstract])) OR (Population at Risk[Title/Abstract])) OR (Populations at Risk[Title/Abstract])) OR (Risk Scores[Title/Abstract])) OR (Risk Score[Title/Abstract])) OR (Score, Risk[Title/Abstract])) OR (Risk Factor Scores[Title/Abstract])) OR (Risk Factor Score[Title/Abstract])) OR (Score, Risk Factor[Title/Abstract])) OR (Health Correlates[Title/Abstract])) OR (Correlates, Health[Title/Abstract])) OR (Social Risk Factors[Title/Abstract])) OR (Factor, Social Risk[Title/Abstract])) OR (Factors, Social Risk[Title/Abstract])) OR (Risk Factor, Social[Title/Abstract])) OR (Risk Factors, Social[Title/Abstract])) OR (Social Risk Factor[Title/Abstract])))

Figure s1 age >60 Meta-analysis sensitivity analysis

Figure s2 BMI >25 Meta-analysis sensitivity analysis

Figure s3 Previous fractures Meta-analysis sensitivity analysis

Figure s4 Low Lumbar BMD Meta-analysis sensitivity analysis

Figure S5 Funnel plot of meta-analysis for age>60

Figure S6 Funnel plot of meta-analysis for BMI≥24

Figure S7 Funnel plot of meta-analysis for Previous fractures

Figure S8 Funnel plot of meta-analysis for Low Lumbar BMD
